# Response: Letter to FKBP5 blockade may provide a new horizon for the treatment of stress‐associated disorders: An in silico study

**DOI:** 10.1002/epi4.12812

**Published:** 2023-09-28

**Authors:** Ali A. Asadi‐Pooya

**Affiliations:** ^1^ Epilepsy Research Center Shiraz University of Medical Sciences Shiraz Iran; ^2^ Jefferson Comprehensive Epilepsy Center, Department of Neurology Thomas Jefferson University Philadelphia Pennsylvania USA

**Keywords:** FKBP5, functional, psychogenic, seizure

To the Editor,

I read with interest the letter in correspondence to our previous publication: “Letter to FKBP5 blockade may provide a new horizon for the treatment of stress‐associated disorders: An in‐silico study” by Dr. Shang et al.^1^ I agree with their final conclusion that “Considering the lack of sufficient evidence‐based evidence, more studies are needed to investigate the relationship between FKBP5 and the identified drugs, which would be a huge challenge.” As we clearly stated in our discussion: “The results of this *in‐silico* study should not be viewed as clinically applicable yet; rather, this endeavor can be simply viewed as an approach to visualize and analyze the existing data to build a hypothesis. Most importantly, the relationship between *FKBP5* polymorphisms and FS should be established in future studies. When such a relationship is established, then a search to find drugs that may provide *FKBP5* blockade would be clinically more meaningful.”^2^ Furthermore, after four steps in our study (searching for molecules, molecular docking results, in‐silico blood–brain barrier permeability analysis, and drug safety data), we concluded that “In the current in‐silico study, we could identify Fluticasone propionate as a good candidate to deliver FKBP5 blockade. Prednisolone and Dexamethasone have also acceptable pharmacological profiles to be used as FKBP5 inhibitors. Some psychiatric drugs (e.g., Mirtazapine, Sertraline, Fluoxetine, and Citalopram) also have acceptable pharmacological profiles to be used as FKBP5 inhibitors.”^2^ In other words, Fluticasone propionate was identified as a good candidate for potential future studies (after the relationship between *FKBP5* polymorphisms and functional seizures [psychogenic nonepileptic seizures/attacks] is established). Other drugs (Prednisolone, Dexamethasone, and some psychiatric drugs, e.g., Mirtazapine, Sertraline, Fluoxetine, Citalopram) had acceptable pharmacological profiles to be used in such studies.

As Dr. Shang et al.^1^ correctly stated, currently, psychotherapy is considered the best treatment option for patients with functional seizures (psychogenic nonepileptic seizures/attacks). However, a large clinical trial (CODES trial) failed to demonstrate that cognitive behavioral therapy has a statistically significant advantage compared with standardized medical care alone for the reduction of monthly seizures in patients with functional seizures (psychogenic nonepileptic seizures/attacks).^3^ Furthermore, a previous meta‐analysis that used data from 13 manuscripts on 228 patients with functional seizures (psychogenic nonepileptic seizures/attacks) showed that 47% of patients achieved seizure control after completion of psychological therapeutic intervention.^4^ On the other hand, a study of the natural history of 69 patients with functional seizures (psychogenic nonepileptic seizures/attacks), who never received a proper psychological treatment, showed that 52% of these patients were seizure‐free in their last follow‐up visit.^5^ Functional seizures (psychogenic nonepileptic seizures/attacks) have significant and devastating effects on patients' lives^6^ and therefore, the scientific community has the obligation to try to discover more efficacious therapeutic options for the treatment of this condition.

## AUTHOR CONTRIBUTIONS

The sole author had responsibility for all parts of the manuscript.

## FUNDING INFORMATION

This research did not receive any specific grant from funding agencies in the public, commercial, or not‐for‐profit sectors.

## CONFLICT OF INTEREST STATEMENT

Honorarium: Cobel Daruo, Actoverco; Royalty: Oxford University Press (Book publication).
